# Cancer Incidence among Heart, Kidney, and Liver Transplant Recipients in Taiwan

**DOI:** 10.1371/journal.pone.0155602

**Published:** 2016-05-19

**Authors:** Kwai-Fong Lee, Yi-Ting Tsai, Chih-Yuan Lin, Chung-Bao Hsieh, Sheng-Tang Wu, Hung-Yen Ke, Yi-Chang Lin, Feng-Yen Lin, Wei-Hwa Lee, Chien-Sung Tsai

**Affiliations:** 1 Graduate Institute of Life Sciences, National Defense Medical Center, Taipei, Taiwan; 2 Biobank Management Center, Tri-Service General Hospital, Taipei, Taiwan; 3 Division of Cardiovascular Surgery, Tri-Service General Hospital, Taipei, Taiwan; 4 Division of General Surgery, Tri-Service General Hospital, Taipei, Taiwan; 5 Division of Urology, Tri-Service General Hospital, Taipei, Taiwan; 6 Department of Internal Medicine, School of Medicine, College of Medicine, Taipei Medical University, Taipei, Taiwan; 7 Division of Cardiology, Department of Internal Medicine and Cardiovascular Research Center, Taipei Medical University Hospital, Taipei, Taiwan; 8 Department of Pathology, Taipei Medical University Shuang-Ho Hospital, New Taipei, Taiwan; 9 Department of Pathology, School of Medicine, College of Medicine, Taipei Medical University, Taipei, Taiwan; 10 Division of Cardiovascular Surgery, Taoyuan Armed Forces General Hospital, Taoyuan, Taiwan; 11 Department and Graduate Institute of Pharmacology, National Defense Medical Center, Taipei, Taiwan; University of Toledo, UNITED STATES

## Abstract

Population-based evidence of the relative risk of cancer among heart, kidney, and liver transplant recipients from Asia is lacking. The Taiwan National Health Insurance Research Database was used to conduct a population-based cohort study of transplant recipients (n = 5396), comprising 801 heart, 2847 kidney, and 1748 liver transplant recipients between 2001 and 2012. Standardized incidence ratios and Cox regression models were used. Compared with the general population, the risk of cancer increased 3.8-fold after heart transplantation, 4.1-fold after kidney transplantation and 4.6-fold after liver transplantation. Cancer occurrence showed considerable variation according to transplanted organs. The most common cancers in all transplant patients were cancers of the head and neck, liver, bladder, and kidney and non-Hodgkin lymphoma. Male recipients had an increased risk of cancers of the head and neck and liver, and female kidney recipients had a significant risk of bladder and kidney cancer. The adjusted hazard ratio for any cancer in all recipients was higher in liver transplant recipients compared with that in heart transplant recipients (hazard ratio = 1.5, *P* = .04). Cancer occurrence varied considerably and posttransplant cancer screening should be performed routinely according to transplanted organ and sex.

## Introduction

Heart, kidney, and liver transplantation are standard procedures for patients with end-stage organ disease. In Taiwan, transplant recipients have excellent outcomes, with 1-year survival rates of 78%–96%. [1] However, cancer incidence is increased in these recipients because of immunosuppressive therapy, medication (analgesic abuse and certain herbal preparations), and viral infections (Epstein–Barr virus [EBV] and hepatitis C and B). Western studies have shown an overall increase in the risk of cancer of 2–10-fold in heart transplant recipients, 2–3-fold in liver transplant recipients, and 2–6-fold in kidney transplant recipients compared with that in the general population. [2–5]

Nevertheless, few population-based studies have been conducted in Asia and are limited mostly to kidney transplantation.[6, 7] The comparison of cancer incidence among recipients of different transplanted organs can clarify the pattern of post transplantation cancer etiology. These evidence-based results can also guide the development of strategies for cancer prevention and benefit high-risk recipients by minimizing the cancer risk.

In Taiwan, the variation in cancer occurrence among different transplanted organs is unclear. Therefore, we estimated the incidence of cancer in heart, kidney, and liver transplant recipients from 2001 to 2012 using the Taiwan National Health Insurance Research Database (NHIRD).

## Methods

### Study population

The 1995 National Health Insurance Act established the National Health Insurance (NHI) program, which is a mandatory single-payer system with the principle of equal access to all health care services. At the end of June 2014, 23 508 577 people (99.9% of Taiwan’s population) were enrolled in the program, and 93% of hospitals and clinics were contracted with the NHI. People with catastrophic illnesses are exempt from copayments to ensure that costly treatment does not impede them from receiving the necessary medical services. Malignant neoplasms and follow-up treatment after kidney, heart, lung, liver, or bone marrow transplant are recognized as catastrophic illnesses in the NHI program. Health care providers are not reimbursed if their submitted medical service claims violate insurance regulations after review and auditing by the National Health Insurance Administration (NHIA). Each year, the NHIA collects data including registration files and original claims data for reimbursement from the NHI program and sorts this information into data files. These deidentified data are sent to the National Health Research Institutes (NHRI) to generate the NHIRD. This study was exempted from institutional review board approval according to the regulations. After customized screenings, the inpatient and outpatient data of heart, kidney, and liver recipients between 2001 and 2012 were extracted from the NHIRD and catastrophic illness dataset.

### Data analysis

Recipients who had received transplantation before 2001 or multiorgan transplantation between 2001 and 2012 and those who were diagnosed with cancer before transplantation were excluded from this study. Cancers were classified according to International Classification of Diseases, Ninth Revision, Clinical Modification (ICD-9-CM) codes 140–208. We also excluded patients who developed cancer within the first 30 days after transplantation. The exception was Kaposi sarcoma, because the incidence rate in the general population is unavailable. Patients were followed until death, subsequent transplantation, their most recent medical record, or the end of 2012—whichever came first. Cancer incidence rates in transplant recipients were compared with Taiwanese general population by using the standardized incidence ratio (SIR). The SIR was defined as the ratio of the observed number of new cases of cancers to the expected number in the general population. The expected number of incident cases was calculated by multiplying the age- and sex-specific incidence rates in the general population obtained from the Taiwan National Cancer Registry for 2001–2011 and the numbers of person-years. The 95% confidence intervals (CIs) for SIRs were calculated by assuming that the observed cancer cases followed a Poisson distribution. We performed additional analyses of the 5 most common cancers with significantly elevated SIRs in all transplanted patients: cancers of the head and neck, bladder, kidney, and liver and non-Hodgkin lymphoma (NHL). The Cox regression model was used to calculate the effects of covariates on the long-term risk of cancer. Risk factors included the recipient’s sex and transplanted organ, both of which were categorical variables. Patient age at transplantation and the calendar year of transplantation were included as continuous variables. Sex, age, calendar year of transplantation, and transplanted organ were used to examine the independent effects on the risk of selected cancers after transplantation. The same analysis was performed separately to investigate the risk of the 5 most common cancers. P values < 0.05 were considered statistically significant. Statistical analyses were performed with the SPSS statistical software, version 18.0 (SPSS, Inc., Chicago, IL, USA).

## Results

### Current statistics

**Fig 1:**
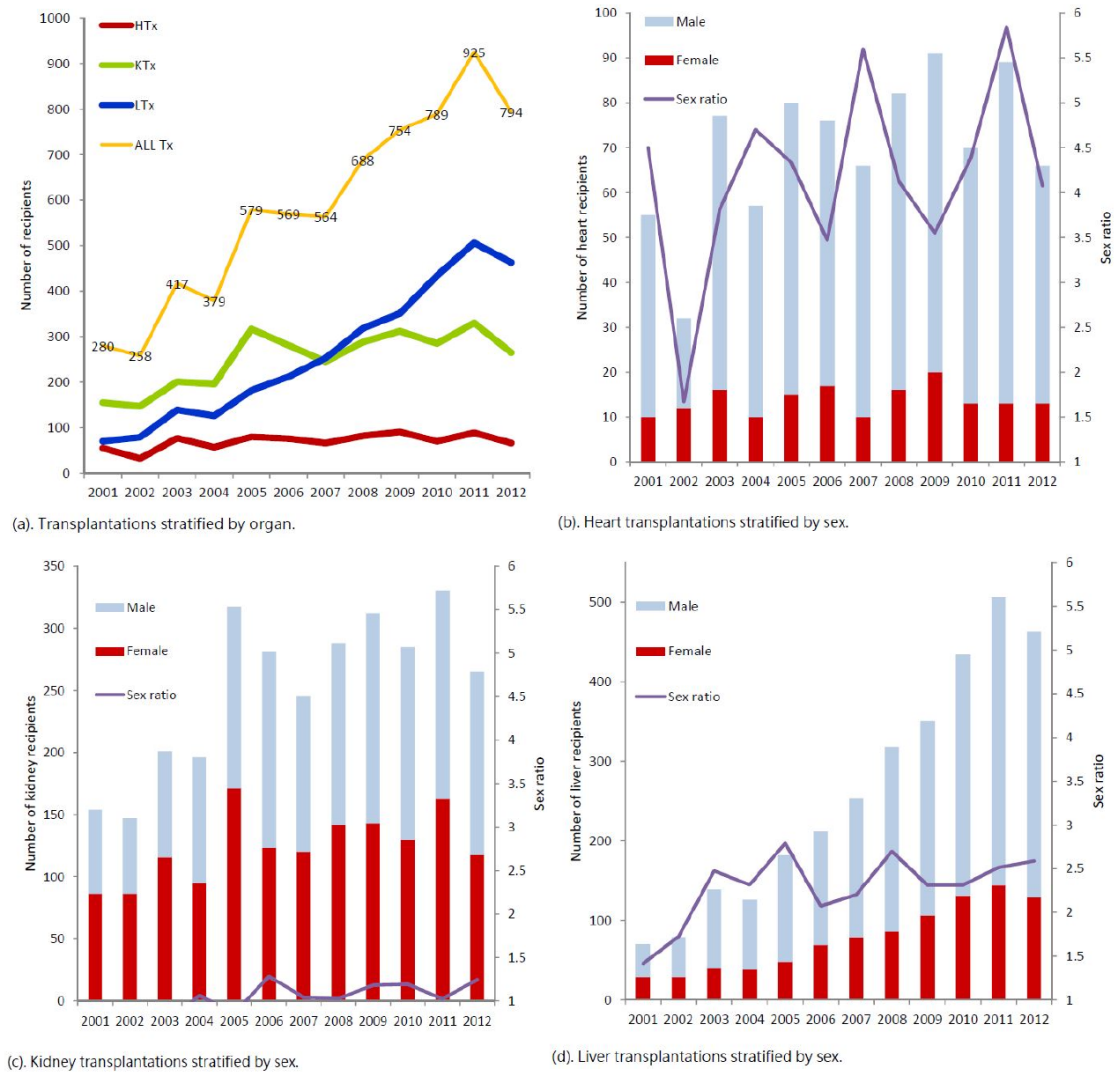
Heart, kidney, and liver transplantations performed during the study period. (a). Transplantations stratified by the organ. (b), (c), and (d). Heart, kidney, and liver transplantations stratified by sex. The purple line indicates the male:female ratio.

From 2001 to 2012, 6995 transplantations were performed in Taiwan, according to the NHIRD (Fig 1). On average, 70 heart, 252 kidney, and 261 liver transplantations were performed annually. The number of heart transplantations remained constant, and the number of liver transplantations increased substantively during the 12-year period. Most of the heart and liver transplant recipients were men (male:female ratio = 3.8 and 2.2, respectively). An equal number of male and female patients received kidney transplants.

### Study cohort

After the exclusion of patients with previous transplants, multiorgan transplants, and cancer diagnosis before transplantation or within 30 days of transplantation, the cohort contained 5396 transplant recipients, comprising 801 (14.8%) heart, 2847 (52.8%) kidney, and 1748 (32.4%) liver transplant recipients (Table 1). The recipients were followed for a total of 22 050 person-years (median individual follow-up duration: 3.4 y). The female recipients had a higher follow-up duration than did the male recipients (median follow-up duration: 3.9 vs. 3.1 y), and the kidney transplant recipients had the highest median follow-up duration of 4.3 years among the organ transplant groups. A total of 326 patients (18.6%) underwent liver transplantation before the age of 20 years; 55.7% (n = 3005) of all transplants occurred in the age group of 40–59.

**Table 1:** Characterisstics of recipients of heart, kidney and liver transplants in Taiwan between 2001 and 2012.

	Heart				Kidney				Liver				ALL			
	No.	%	PY	%	No.	%	PY	%	No.	%	PY	%	No.	%	PY	%
Total	801		2808.51		2847		13369.24		1748		5879.44		5396		22057.19	
Sex																
Male	653	81.52	2225.88	79.25	1435	50.40	6386.62	47.77	1139	65.16	3735.61	63.54	3227	59.80	12348.10	55.98
Female	148	18.48	582.63	20.75	1412	49.60	6982.62	52.23	609	34.84	2143.83	36.46	2169	40.20	9709.09	44.02
Age at transplant, y																
0–9	14	1.75	50.83	1.81	17	0.60	65.35	0.49	269	15.39	1417.96	24.12	300	5.56	1534.14	6.96
10–19	43	5.37	163.82	5.85	113	3.97	589.75	4.41	57	3.26	207.85	3.54	213	3.95	961.42	4.36
20–29	58	7.24	223.93	7.99	321	11.28	1847.57	13.82	47	2.69	155.60	2.65	426	7.89	2227.10	10.10
30–39	110	13.73	437.51	15.62	612	21.50	3143.68	23.51	122	6.98	424.66	7.22	844	15.64	4005.85	18.17
40–49	173	21.60	619.76	22.13	849	29.82	4182.45	31.28	426	24.37	1438.48	24.47	1448	26.83	6240.70	28.30
50–59	253	31.59	855.51	30.54	718	25.22	2872.62	21.48	586	33.52	1680.04	28.57	1557	28.85	5408.17	24.52
≥ 60	150	18.73	449.77	16.06	217	7.62	669.99	5.01	241	13.79	554.86	9.44	608	11.27	1674.62	7.59

Abbreviations: PY, person years.

### Cancer risk according to the transplanted organ in comparison with the general population

**Fig 2:**
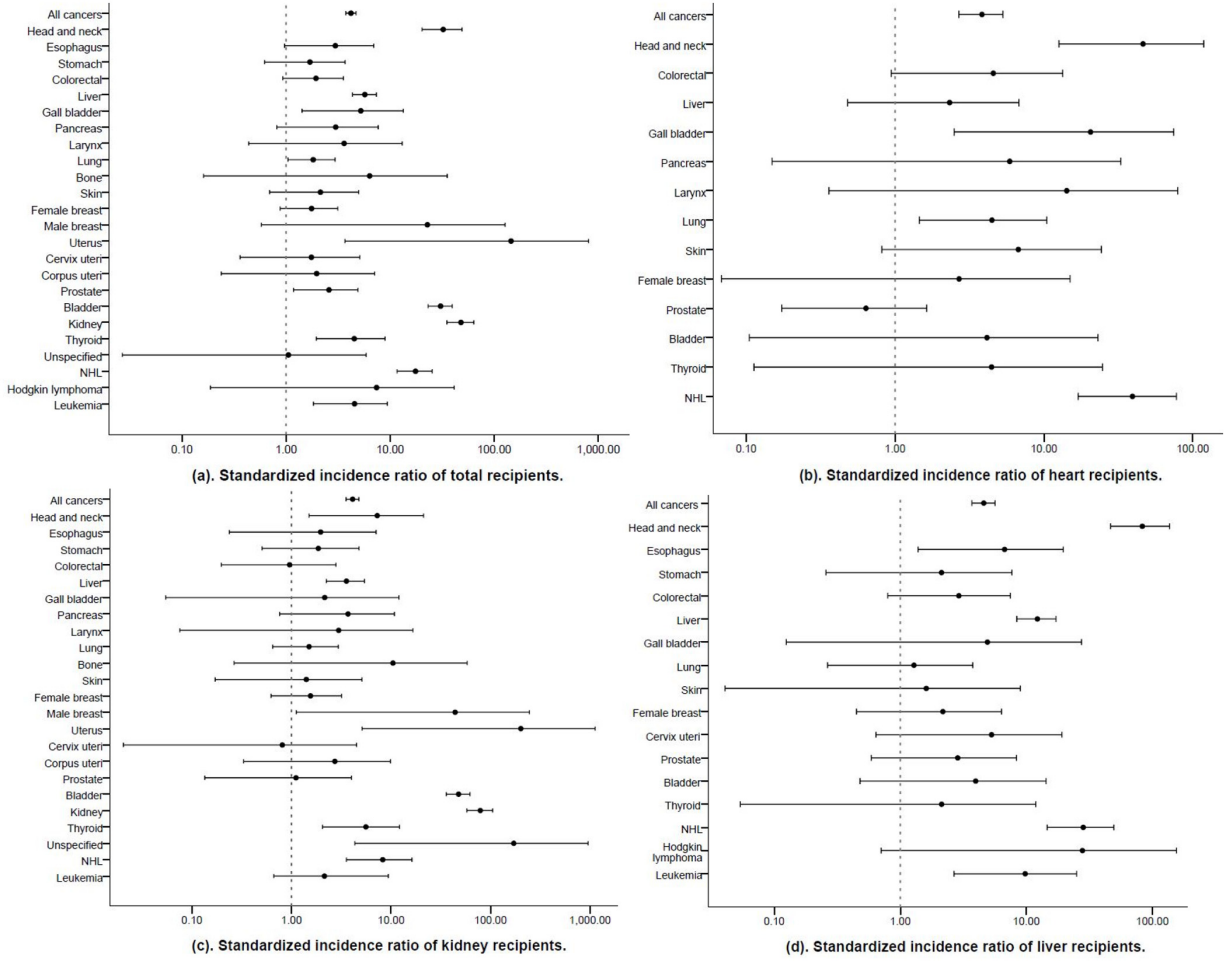
Risk of cancer among Taiwanese recipients. (a). Cancer risk for total recipients. (b), (c), and (d). Cancer risk for heart, kidney, and liver recipients.

Of the 5396 transplant recipients, 310 developed malignancies during the follow-up, corresponding to a 4-fold higher cancer risk compared with that of the general population (SIR = 4.2, 95% CI 3.8–4.7). Compared with the general population, the risk of any cancer was 3-fold higher in the heart recipients (SIR = 3.8, 95% CI 2.7–5.3) and 4-fold higher in the kidney (SIR = 4.1, 95% CI 3.5–4.8) and liver (SIR = 4.6, 95% CI 3.7–5.6) recipients (Table 2, Fig 2).

**Table 2:** Standardized incidence ratios (SIRs) of cancer sites among heart, kidney, and liver between 2001 and 2012, NHIRD.

		Heart			Kidney			Liver			Total		
Cancer site	ICD-9-CM	O	E	SIR	O	E	SIR	O	E	SIR	O	E	SIR
All cancers	140–208	36	9.38	3.84	184	44.66	4.12	90	19.64	4.58	310	73.66	4.21
Head and neck	140–149	4	0.09	46.32	3	0.41	7.28	15	0.18	82.85	22	0.68	32.38
Esophagus	150				2	1.02	1.96	3	0.45	6.70	5	1.68	2.98
Stomach	151				4	2.15	1.86	2	0.94	2.12	6	3.54	1.69
Colorectal	153–154	3	0.66	4.57	3	3.13	0.96	4	1.38	2.91	10	5.16	1.94
Liver	155	3	1.29	2.32	22	6.16	3.57	33	2.71	12.20	58	10.15	5.71
Gall bladder	156	2	0.10	20.58	1	0.46	2.16	1	0.20	4.90	4	0.76	5.23
Pancreas	157	1	0.17	5.90	3	0.81	3.71				4	1.33	3.00
Larynx	161	1	0.07	14.23	1	0.34	2.98				2	0.55	3.62
Lung	162	5	1.12	4.48	8	5.33	1.50	3	2.34	1.28	16	8.78	1.82
Bone	170				1	0.10	10.47				1	0.16	6.35
Skin	173	2	0.30	6.73	2	1.42	1.41	1	0.62	1.60	5	2.34	2.14
Female breast	174	1	0.37	2.69	7	4.51	1.55	3	1.38	2.17	11	6.26	1.76
Male breast	175				1	0.02	44.16				1	0.04	22.84
Uterus	179				1	0.00	201.97				1	0.01	145.45
Cervix uteri	180				1	1.24	0.81	2	0.38	5.29	3	1.72	1.75
Corpus uteri	182				2	0.73	2.73				2	1.02	1.97
Prostate	185	4	6.28	0.64	2	1.80	1.11	3	1.05	2.85	9	3.48	2.58
Bladder	188	1	0.24	4.15	55	1.15	47.78	2	0.51	3.95	58	1.90	30.56
Kidney	189				47	0.59	79.04				47	0.98	47.94
Thyroid	193	1	0.22	4.45	6	1.07	5.59	1	0.47	2.12	8	1.77	4.52
Unspecified	199				1	0.01	171.41				1	0.95	1.05
NHL	200, 202, 203	8	0.20	39.33	8	0.97	8.26	12	0.43	28.21	28	1.60	17.54
Hodgkin lymphoma	201							1	0.04	27.79	1	0.14	7.40
Leukemia	204–208				3	1.40	2.14	4	0.41	9.76	7	1.54	4.55

Abbreviations: O, observed numbers of cases; E, expected numbers of cases.

The estimated risks of head and neck cancer and NHL were substantially increased among all the recipients, regardless of transplantation type. The risk of liver cancer was significantly elevated in both kidney (SIR = 3.6, 95% CI 2.2–5.4) and liver (SIR = 12.2, 95% CI 8.4–17.1) transplant recipients, and 3 incident cases of liver cancer were observed in the heart recipients.

The cancer risk according to the transplanted organ varied. The risks of gallbladder (SIR = 20.6, 95% CI 2.5–74.3) and lung cancer (SIR = 4.5, 95% CI 1.5–10.5) were significantly increased only in the heart recipients. Three incident cases of male breast cancer, uterine cancer, and an unspecified cancer occurred in the kidney recipients. The thyroid cancer (SIR = 5.6, 95% CI 2.1–12.2) risk was significantly elevated only in the kidney recipients. The risks of bladder (SIR = 47.8, 95% CI 36.0–62.2) and kidney cancer (SIR = 79.0, 95% CI 58.1–105.1) were observed in kidney recipients. Esophageal cancer and leukemia were also observed in the kidney and liver recipients; however, both risks were significantly increased in only the liver recipients (SIR = 6.7, 95% CI 1.4–19.6 and SIR = 9.8, 95% CI 2.7–25.0, respectively).

### Cancer risk comparison

Table 3 presents the risk of selected cancers for the transplant recipients. The table presents estimated hazard ratios (HRs) for the occurrence of head and neck, liver, bladder, and kidney cancer; NHL; and all cancers.

**Table 3:** Risk factors for selected cancer in transplanted recipients.

	Head and neck		Liver		Bladder		Kidney^1^		NHL		ALL	
	HR	p-value	HR	p-value	HR	p-value	HR	p-value	HR	p-value	HR	p-value
Age at transplant (years)	1.03	0.07	1.05	<0.001	1.06	<0.001	1.04	<0.001	0.97	<0.01	1.04	<0.001
Sex												
Male	1	(ref)	1	(ref)	1	(ref)	1	(ref)	1	(ref)	1	(ref)
Female	0.18	0.02	0.53	0.03	2.29	<0.01	3.52	<0.001	0.70	0.40	1.01	0.95
Calendar year of transplantation	1.24	0.06	0.90	0.03	0.94	0.23	0.87	0.02	0.86	0.04	0.95	0.03
Transplanted organ												
Heart	1	(ref)	1	(ref)	1	(ref)			1	(ref)	1	(ref)
Kidney	0.25	0.07	2.38	0.16	11.90	0.01			0.23	<0.01	1.31	0.15
Liver	2.13	0.18	7.05	<0.01	1.08	0.95			0.55	0.23	1.52	0.04

HR = hazard ratio.

^1^The kidney cancers were all contributed from kidney transplantations and the model was adjusted for age at transplant (years), sex and calendar year of transplantation.

The results of the Cox regression model indicated that older age at transplantation, early transplantation, and liver transplantation were risk factors for any cancer. For liver, bladder, and kidney cancer, the hazard of occurrence increased with age; however, younger recipients had an increased risk of NHL. Male recipients had an increased risk of cancers of the head and neck and the liver; however, female recipients had a significant risk of bladder and kidney cancer, though they had undergone kidney transplantation. Patients had a significantly lower risk of liver and kidney cancer and NHL in the current year. Recipients of heart transplants and those with transplantation at a younger age or early transplantation had an increased risk of NHL.

## Discussion

This Taiwanese, population-based cohort of more than 5000 organ transplant recipients, who were followed for 12 years, enabled the determination of variations in cancer risk according to the transplanted organ, age at transplantation, and sex.

Previous Asian population-based studies comparing the risk of cancers between organ transplant recipients and the general population have focused on recipients of specific organ transplants,[6, 8–10] with few studies having compared the risk of cancer in heart, kidney, and liver recipients. Compared with the general population, the risk of cancer increased 3.8-fold after heart transplantation, 4.1-fold after kidney transplantation, and 4.6-fold after liver transplantation, and this risk increased by an average of 4.2-fold in all transplant recipients. The most common cancers after transplantations in Taiwan were cancers of the head and neck, liver, bladder, and kidney and NHL. Male recipients had an increased risk of cancers of the head and neck and the liver; however, female kidney recipients had a significant risk of bladder and kidney cancer.

Our study presents population-based evidence of a significantly higher risk of any cancer in liver recipients than in both heart and kidney recipients after adjustment for age at transplantation, sex, and transplant year.

We have observed increased risks of NHL and cancers of the head and neck, gallbladder, and lung in heart recipients and confirmed prior population-based evidence of increased risks of lip and oral cancer among heart transplant recipients, [2, 3, 5, 11] with an SIR of 46.3 for cancers of the head and neck compared with the general Taiwan population. Viral infection, immunosuppressive therapy, or drug side effects may increase the likelihood of oral ulcers or tongue edema in transplant recipients.[12–16]

Our data of lung cancer and NHL in the heart transplant recipients was consistent with western studies.[2, 3, 5, 11, 17] Smoking is associated with lung cancer[18]; however, it does not play a significant role in the incidence of lung cancer among Spanish patients. [19] The risk of NHL among transplant recipients is related to the aggressiveness of the immunosuppressive regimen.[20] EBV infection plays a crucial role in posttransplant lymphoproliferative disorder in pediatric heart transplant patients,[21, 22] and an EBV-negative serostatus is associated with an increased risk of NHL in heart transplant patients (HR = 3.6, 95% CI 1.1–11.3; *P* = .031).[23] We observed a 20-fold increased risk of gallbladder cancer in the heart transplant recipients. The risk of gallbladder cancer has been rising among kidney transplant recipients, [9, 24, 25]but similar to general population in US.[26]

We also confirmed liver transplantation was associated with an increased risk of NHL, [3–5, 11, 17, 27, 28] leukemia, and cancer of the head and neck,[3, 5, 11, 28] esophagus,[28] and liver.[3, 17] Alcohol-related cirrhosis in patients with a history of tobacco abuse before liver transplantation may cause the development of head and neck and esophageal cancer.[28–30] The current study demonstrated that in liver recipients, older age and being male were risk factors for liver cancer; this finding was consistent with US registry data.[31] Among the liver recipients, the risk of NHL was virtually unaffected by EBV serostatus; however, it was much higher than that observed in EBV-positive kidney or heart transplant recipients.[23] Because of the scarcity of leukemia after liver transplantation, cases, including donor-derived acute myeloid leukemia, pediatric patient, therapy-related chronic myelomonocytic leukemia, and hepatitis B virus-associated cirrhosis, have been reported. [32–36] An early research has shown parvovirus B19 may be relevant in the pathogenesis of acute leukemia.[37] Nevertheless, the risk of leukemia after liver transplantation remains unclear.

Our results of kidney transplantation concur with prior population-based evidence on the risks of NHL and cancers of the kidney, bladder, and thyroid[3, 5, 6, 24] but not cancers of the head and neck [3, 6] or liver.[24] We found that female kidney recipients had a higher SIR for bladder cancer (SIR male = 20.7 and SIR female = 113.2; data not shown), supporting previous Asian researches.[6, 7, 9, 10] The binding of female hormones to their receptors exhibits rapid bladder cancer progression in women[38–40]; however, few Western studies have examined the reproducibility of sex differences in bladder cancer after kidney transplantation. [41] Additional studies are warranted to clarify such associations. Female kidney recipients have a higher risk of urinary tract infections than do male recipients. [42] Papillary thyroid carcinoma accounts for more than 88% of all thyroid cancers in Taiwan. [43] Viral infections have been associated with thyroid cancer progression. [44–46] The high incidence of thyroid disorders among kidney recipients necessitates that cancer-related examinations be performed routinely in patients with viral infections and renal disorders. [47, 48]

In Taiwan, liver and oral cancers account for the third and sixth highest incidences of cancer in the general population, and the incidence is particularly high in men. [43] The prevalence is high in patients with chronic hepatitis B virus (HBV) infections (13.7%). [49] Current alcohol consumption, smoking, and self-medication are factors that are correlated to the high cancer prevalence in adults with HBV or hepatitis C virus infections. [50] Heavy alcohol consumption significantly increased the risk of hepatocellular carcinoma in patients with HBV-related cirrhosis. [51] Betel quid use (with or without tobacco addition) has been confirmed as a major independent risk factor for oral and oropharyngeal cancers. [52] Betel quid chewing was observed in 90% of patients with oral cancer. [53] In a cross-sectional study, the prevalences of alcohol consumption, betel quid chewing, and smoking in men were 53.2%, 32.7%, and 50.2%, respectively. [54] Hepatitis virus infections, betel quid chewing, alcohol consumption, and smoking may partially explain the increased risk of liver and oral cancers among male recipients.

Skin cancer after transplantation has been the major concern identified in western studies.[3, 5, 11, 55] This was not observed in our study. Continual sun exposure from childhood is a risk factor for squamous cell carcinoma (SCC); however, the risk varies according to sex. [56] In arseniasis-endemic areas, standardised mortality ratios are significantly high for liver, lung, and bladder cancers. Thus, cutaneous SCC and basal cell carcinoma (BCC) are more strongly associated with arsenic exposure than with sun exposure. [57, 58] Because skin cancer accounts for the 10th highest incidence of cancer in the general population in Taiwan [43] and a study conducted in southern reported no incidence of SCC or BCC after renal transplantation, [59] genetic factors can influence the ethnic variance observed in the occurrence of skin cancer.

Calendar year of transplantation had been reported as protective factors[11, 55], risk factors[2, 5] and non-significant factors[3, 4]. Because these results published from different countries, transplant organs, periods of calendar year of transplantations and cancers, the impact of calendar year of transplantation remains uncertain. Therefore, the association of age and calendar year of transplantation also needs further studies to clarify.

Though this 12-year study period might not sufficient for certain cancer occur, [60, 61] cancers of the head and neck, liver, bladder, and kidney and non-Hodgkin lymphoma after transplantation in Taiwan alert health professionals to the importance of cancer-related screening and behavior risk factors during follow up.

Reviews by the NHIA and government regulation ensure the quality of the NHIRD, which covers almost the entire population of Taiwan. Furthermore, because of the demanding nature of immunosuppressive therapy and copayments are waived for patients with catastrophic illnesses, it is unlikely that transplant recipients would travel abroad or that our estimates would be biased by loss to follow-up. However, patients traveling abroad for transplantation may have led to our study underestimating the cancer incidence rates. By the nature of NHIRD, the lack information of behavior risk factors, donor–recipient individual and matching data [62–66] and cancers arising from the native organs or the transplant limits the elaboration of this research.

In conclusion, this Asian population-based study showed an elevated cancer risk among transplant recipients. Cancer occurrence varied considerably and posttransplant cancer screening should be performed routinely according to transplanted organ and sex. Our findings encourage further studies on oncogenic mechanisms of organ transplantation in the future.
